# A survey of elite and pre-elite athletes’ perceptions of key support, lifestyle and performance factors

**DOI:** 10.1186/s13102-021-00393-y

**Published:** 2022-01-03

**Authors:** Lauren Burns, Juanita R. Weissensteiner, Marc Cohen, Stephen R. Bird

**Affiliations:** 1 School of Health and Biomedical Sciences, RMIT University, Bundoora, VIC 3083 Australia; 2 Sport Development, New South Wales Office of Sport, Sydney Olympic Park, NSW 2127 Australia; 3 Extreme Wellness Institute, 13 Somers Rd, Warrandyte, VIC 3113 Australia

**Keywords:** Olympic, Paralympic, Holistic, Sport, Psychology, Athlete, Wellbeing

## Abstract

**Background:**

Success at the elite level in sport is often attributed to physical prowess, technical skill, and mental attitude. However, underpinning these factors are various lifestyle, support and social factors that may contribute to successful performance, but which may be absent from athlete development programs.

**Methods:**

An online survey was used to investigate athlete perceptions of lifestyle practices and support services amongst 135 Australian Olympic, Paralympic, National, and state-level athletes across 25 Olympic sports.

**Results:**

International athletes perceived psychological skills and attributes, along with strong interpersonal relationships as vital to their success, and they also rated ‘Recovery practices’ as very important and made extensive use of available support services. These athletes also indicated that they would have liked access to these services earlier in their careers, a wish that was reiterated by the sub-elite athletes. Furthermore, athletes wanted greater knowledge, mentoring, and autonomy earlier in their careers, and the importance of ‘athlete wellbeing’ as well as ‘athletic performance’ was evident in a number of contexts.

**Conclusions:**

An athlete development system into which these are included may assist in generating an environment that facilitates athlete success, repeated podium performances, retain athletes in high-performance sport for longer, encourage human-flourishing, wellbeing and smooth transitions for retiring athletes.

## Background

The pursuit of excellence requires elite athletes to have an intense, myopic focus on their sport, yet they must also contend with the demands of daily life [1]. These psycho-social and ‘well-being’ aspects can impact upon their performance, as well as the athlete’s pursuit of sporting excellence having the potential to affect their well-being in both positive and negative ways. Athlete wellbeing and performance may be influenced by family, friends, professional and personal-development programs, spirituality and social-connections [1, 2]. In conjunction with this, the strengthening of life-skills is essential for developing resilience [1], which is needed in both the athlete’s sporting and non-sporting environments. It is therefore a responsibility of those overseeing athlete development to maintain a duty-of-care that ensures the wellbeing of athletes throughout their sporting life and beyond. In accordance with this, international sporting bodies advocate an holistic approach to the athletic profile, and one that incorporates lifestyle and system level factors that are required for world-class sporting performance [2]. Yet despite this, key lifestyle, relational, psycho-social, performance and recovery practices are commonly overlooked in athlete development and support systems.

Our previous research into this topic, involved interviewing World and Olympic Champions, from which we identified four key ‘Psycho-social and Lifestyle’ themes: (1) psychological skills and attributes; (2) interpersonal relationships; (3) performance factors/strategies; and (4) lifestyle practices [3]. These findings suggested that achieving world-class podium-level performance is multi-dimensional, involving not just ‘talent’ and training, but key psycho-social factors that athletes believe to be of paramount importance [3, 4]. From this we deemed that success both within sport and transition out of sport requires an holistic approach to athlete development that includes a complimentary mix of wellbeing, lifestyle practices, performance strategies, psychological attributes, education, and supportive interpersonal relationships [5]. Pre-elite athletes have been identified as the most vulnerable population coming through athlete development pathways and are subject to drop-out and burn-out [6]. Therefore, it is essential to quantify and understand gaps in the system and provide support, screening, and education platforms that can assess a developing athlete’s competence, facilitate sport-specific education, and implement interventions to maximise an athlete’s longevity, performance-success, and transition to retirement. In pursuit of this we wished to further understand the perceived significance of these key factors for athletes within the athlete development pathway, as this would help to inform the evolution of an holistic approach to athlete support and development. Additionally, the findings could be used to inform the development of a valid and reliable screening tool for the assessment of a ‘developing’ athlete’s support, access and awareness of key psycho-social and lifestyle factors.

The aims of this study were therefore to gain a deeper understanding of the prevalence, utilisation and perceived impact of key lifestyle, relational, and support practices of athletes across a variety of sports at different stages of their development pathway. A secondary aim was to identify any differences in these between athletes of different levels of achievement; able-bodied (AB) athletes and athletes with disabilities (AwD); male and female athletes; and sports in which winning was determined by centimetres, grams and seconds (cgs) and non-cgs sports [7, 8].

## Methods

### Online survey

An online survey was designed to: (1) collect data on the type, frequency and quality of lifestyle aspects and practices used by athletes (psychological skills/attributes, interpersonal relationships, support, recovery, sleep, extra-curricular activities, and relaxation); (2) determine athlete knowledge and awareness of the potential impact of these psychological skills/attributes, support services and lifestyle practices; and (3) compare these responses between athletes with different demographics and sporting achievement. The survey was informed by the major themes identified in our previous research and entailed 35 questions with four written response questions [3]. The survey was administered on-line using QualtricsXM software. To ensure reliability, a pilot survey was completed by five retired elite athletes who had competed at senior international level. These athletes completed the draft survey twice and their responses were assessed for reliability (individually and collectively), as well as for the refinement of the questions.

### Recruitment, participants and procedures

Australian athletes in Olympic and Paralympic sports, who had competed at state level or above in the past 10 years, were invited to participate. Participants were recruited via an email sent from one of the following: National Sporting Organisations (NSO’s); State Institute and Academies of Sport; State Sporting Organisations; and the Australian Olympic and Paralympic Committee. This email contained an invitation to participate in the study, a participant information form, and a link to the online survey. Athletes aged over 18 years were emailed this information from their relevant sporting body (listed above), whilst for athletes aged less than 18 years, the email was sent to their parents/guardians with a request to forward the information and survey link to the athlete. Within this email it was stated that parental consent would be inferred, if they forwarded the survey to their child, and that child assent to completing the survey would be inferred from their submission of the completed survey. The survey was completed ‘anonymously’, however, whilst minimal personal and identifying information was asked for in the survey, the researchers acknowledged that in a few cases there was a risk that the sporting achievements reported by the athlete, such as individual medals in international competitions in a specific sport, could enable the researchers to identify the respondent. Participants were informed of the study’s purpose and informed that no identifying information about them would be used when reporting the results of the study. Participation was voluntary, and only the researchers had access to the data.

### Analysis

Data were exported from Qualtrics (Qualtrics Labs Inc.) into SPSS (v 25) and cleaned. Cleaning involved: removing all participants who did not meet the inclusion criteria (see below) and the removal of data from partially completed surveys.

In the analysis, the FTEM (Foundation, Talent, Elite, Mastery) developmental framework was used to classify the achievement levels of the athletes [7, 8]. These classifications are as follows: M = Attained Multiple Podiums (medals) in Senior International Events; E2 = Attained a Single Podium (medal) in a Senior International Event; E1 = Competed at Senior International level (but no medals at Senior International level); T4 = Competed at Junior International or Senior State or Senior National level; T3 = Practicing/Achieving at state level. ‘Foundation’ level athletes, as defined in the model, as not having competed at state level or above, were not intentionally recruited or their data included in the survey, since the focus of the research was on the issues facing athletes at a higher level in the development pathway. Athletes meeting the criteria were then categorised into two groups for comparison: (1) M and E2 athletes who had achieved at least one International Podium (IP) and (2) E1, T4 and T3 athletes who had not achieved an International Podium (n-IP). Additional classifications were: Able-Bodied (AB), and Athletes with a Disability (AwD).

A total of 331 participants commenced the survey. One-hundred and ninety-six were excluded due to not meeting the inclusion criteria, such as their event not being an Olympic sport, or having competed more than 10 years ago. Incomplete responses and duplicates were also removed. The responses of 135 athletes were therefore included in the analysis. Quantitative data were checked for statistical violations and SPSS (v25) statistical software were used to analyse the data. Descriptive (mean and SD) and comparative (ANOVA and t-tests) statistics were used for parametric data, whilst frequencies, percentages and chi-square analyses were used for categorical data.

The responses to the qualitative questions (written response questions) were categorised, using Strauss and Corbin’s method of grounded theory into headings consistent with the higher order themes identified from previous research [9, 10], these being: Psychological Skills and Attributes; Interpersonal Relationships; Performance Factors/Strategies; and Lifestyle Practices [3].

## Results

The responses of 135 athletes (58 male and 77 female) were included in the analysis (see Table 1 for athlete demographics). Twenty-five different Olympic and Paralympic sports were represented. All quantitative data are presented as mean ± SD, unless otherwise stated. Forty-five percent of athletes described their Daily Training Environment (DTE) as centralised, with services such massage, physiotherapy provided through sports institutes or similar, 48% were de-centralised, and 7% selected ‘other’. Forty-four percent of athletes had a scholarship with a state sporting institute, 3% with a regional academy, 15% with a professional club, and 37% indicated ‘other’.

**Table 1:** Athlete FTEM classification and AB/AwD athletes

Sport	Achievement				Total
	n-IP (E1/T4/T3)		IP (M/E2)		
	AB	AwD	AB	AwD	
Total	83	10	30	12	135

Three independent-sample t-tests were conducted to compare the training hours per week of IP and n-IP athletes at time periods: (i) throughout the year, (ii) lead-up to a competition, and (iii) during competition. The mean training hours of IP-athletes throughout the year was higher than for IP-athletes (19.7 v 14.1 h; *p* < 0.005). Training hours in the lead up to competition and during competition were not different between these groups. However, female athletes reported training more hours than male athletes in the weeks leading up to competition (18.5 v 15.4 h) and during competition (11.7 v 8.6 h) (*p* < 0.05). Further analysis using ANOVA did not reveal this difference to be associated with any other factor, and it cannot therefore be explained based on the type of sport or level of sporting achievement.

Time spent away at international events in the previous year was significantly higher for IP-athletes (10.9 v 5.2 weeks; *p* < 0.001) while time spent away from home at domestic events were similar (IP 6.2 v n-IP 8.1 weeks). Factors such as male vs female and AB vs AwD were not associated with any differences in training or travel time.

The results and findings are presented under the established themes of: Psychological Skills and Attributes; Interpersonal Relationships; Performance Factors/Strategies; and Lifestyle Practices. With illustrative responses from the athletes presented in Table 2.

**Table 2:** Representative quotes in response to questions:

***If you have achieved a medal at a benchmark event—why, in your opinion did you succeed compared to your less successful counterpart***
*Superior self-regulation*
Train hard, believe in yourself, maintain perspective and be in the moment. (IP)
*Strong work ethic*
We set out the goal to win, and everything we did, every training session, every meeting, every recovery session, was all very much focused on that and it was consistently at the forefront of our mind. That melted away any complaints, of too much hard work, or too boring meetings, or being tired, it drove us to DO the right things, even if and when we didn't FEEL like it, because the goal was bigger than any obstacle. (n-IP)
*Effective coping strategies and positive mindset*
Confidence in the process and the training (IP)
***Responses in the themes of: superior self-regulation; intrinsic motivation; and effective coping strategies and positive mindset***
*Superior self-regulation*
Self-reflection. To know the good in the bad, and the bad in the good and to subulate each into your performance. For me, being an athlete is about the movement of your own performances throughout your career. Incorporate your previous performances into how you will perform in the present. (n-IP)
*Intrinsic motivation*
You have to be tough. And I also feel that if you are able to cultivate an internal motivational complex, you are far more likely to be successful. For example, if your motivation come from within, you are more likely to train harder every session, miss less training sessions and put yourself in the necessary uncomfortable situations without prompting. (n-IP)
*Effective coping strategies and positive mindset*
Mental training is the key to success, invest time in this. (IP)
***Examples of responses from athletes concerning the importance of interpersonal relationships***
Establish a support team around the athlete as early as possible. These support members will then be able to guide the athlete and help develop the mental framework so that the athlete exhibits better thoughtful action and awareness, as suitable for the individual athlete and their individual sport. (IP)
Find a great coach who understands you and who you are as a person as well as who you are as an athlete, and the ways to support you best. Find the best people to support you and guide you and to train with as early as possible so you don't miss out on valuable time, or waste time learning poor techniques/skills/ideas/strategies. Work hard so you have no regrets. (IP)
To insure sport/life/school balance. The earlier you can see a counsellor or sport psych is important, particularly if you are competing at a high-level during puberty. Insuring support is always in place at training, not always sporting related but social support for LGBTQI and gendered related issues as the dynamic at training with a group of young people can bring up a lot of issues/concerns in this area. (n-IP)
Support from friends and family is crucial, let them know you appreciate it. (n-IP)
Ensuring that those who are around young/newer athletes in a sport have the athletes’ best interests at the heart of their core priorities. I witnessed first-hand in two sports parents/coaches living their goals and expectations through young athletes. It often destroyed the athlete and most certainly their passion for the sport. (n-IP)
Focus and getting on the same page with my teammate…. emotional resilience was strong. Purposeful practice and visions. (IP)
***What could be done better to educate and empower athletes regarding their knowledge and effective usage of athlete and lifestyle strategies?***
*Get advice from experienced/retired athletes new sub-theme*
Other elite athletes being available to share their experiences and be available to meet with developing athletes to help guide and mentor them. (IP)
Have more top athletes visiting younger athletes in sport to give them a better perspective of life in their sport. (n-IP)
*Education (for athletes and coaches) New sub-theme*
More podcasts/e-Learning modules sent to athletes. (IP)
Coaches set up the program from the beginning to incorporate the WHOLE plan, not just the technical plan! (IP)
*Effective use of SMSS*
More balanced lifestyles, a more scientific approach to elite performance, psychological/nutritional services etc. etc. is relatively new, at least its significant importance to performance has only recently been realised. (IP)
*Maximising training and performance opportunities*
Opportunities that provide greater flexibility to undertake education and work, in a capacity that still allows training and travel to work as expected by sporting bodies/ SIS/SAS and sponsors etc. (n-IP)
Educational videos and support from officials. (n-IP)
Subtle information throughout each training session rather than hour long info sessions. (n-IP)
***A summary of responses to questions concerning – ‘lifestyle Practices’, ‘advice and strategies for young athletes’, and what they would have done differently***
*Lifestyle practices*
The importance of not just 2 h at training but the other 22 and how you utilise them. (n-IP) Emphasising the importance of sport/life balance, particularly in sports with limited or non-existent support mechanisms and difficult sporting politics. (n-IP)
*Importance of time out*
Reminder that athletes need downtime as well from high intensity schedules (n-IP)
Honestly finding time to relax is key. Having a healthy balance between work, training and life is essential. Alongside that, realising that this is a marathon not a sprint. It’s going to take a long time and you’re going to fail sometimes, but never be afraid of failure. Also find a supportive and encouraging partner. Because life as a HP athlete is a selfish one and it’s important that family and spouses realise that. (n-IP)
*Balance*
Conversations and advocate a balanced life. (IP)
Don't forget to live. This will help you contextualise yourself in your sport as it relates to your life. Perhaps, then you will regain an enjoyment for your sport. (n-IP)
Plan school, rest and training. Not the other way around. Career/school is important. (n-IP)
*Nutrition and hydration*
Eat food to fuel your body. Whole foods are the best for you and if you are eating a balanced diet you don't need supplements. (n-IP)
Meal preparation is key to success. During competition you need to work out the snacks and meals that will best fuel you for performance. (IP)
Younger athletes’ bodies are continuously growing and developing, therefore avoid dieting or starving strategies to make weigh-ins. In essence, chose divisions that correspond to your natural weight rather than lower/higher weight classes that require extreme weight fluctuations. (n-IP)
*Balance in lifestyle (A new sub-theme)*
A well-rounded person makes a good athlete. You must love what you do but also live a fulfilling and nourishing life. If that means a drink of alcohol or a block of chocolate here and there, it will not be detrimental to your performance. Eat lots, have fun! (n-IP)
Maintain a good balance between sport and other parts of life—otherwise it is easy to get burned out. Spend time to plan out each day to make sure you have time for everything you need to do. (n-IP)
Always keep in mind that even though you love the sport and you want to be the best in the business, you have a life outside of it. Learn another skill, socialise and always think about the future. Have the support of your family is important and have a team behind you. (IP)
Keep it fun and have something else to fall back on. Sport is not life. (n-IP)
*Planning and goal setting (A new sub-theme)*
Focusing and setting goals and having a plan but also making sure you don’t overwork yourself and allow yourself to unwind and relax and recover between training and competing. (IP)
Know the importance of being an elite athlete and eating, planning and structuring your day and overall year to make you the best performer. (n-IP)
Set your goals and then set mini goals within those goals. Don’t give up and keep moving forward. Worry about how what you do makes you feel, not anyone else. (n-IP)
*Know your ‘Why’ (New sub-theme)*
My advice to make sure you understand your driver. What drives you to do the sport. Knowing your why? When you know why you do something, the driver, energy, passion, dedication and desire to succeed is very clear. When you don't know your why, then it’s very hard to commit, pursue and persist with elite level sport. When hardships, sacrifices, adversity and everything else that gets thrown at athletes, the WHY needs to be clear so the pull and the want to keep moving forward with focus it automatic and STRONG when road blocks appear. The WHY creates a clear vision!! (IP)
Find your 'why'. (n-IP)
Know why you are doing it. Understand how you are going to do it. Do it. (IP)
*Take control/know yourself (new sub-theme)*
Ensure you have the right people around you for 'YOU'!
Focus on who you want to be and what you want to achieve, don’t let anyone change you into someone you don’t want to be. Stay true to yourself, know your emotions and feelings, know your body
Don’t be afraid to seek help
Be organised. Take time to be by yourself when you need it. Reach out for support. Be fit. Be healthy. Be happy
You need to learn how to have tough skin in any sport and how to have self-motivation be disciplined enough to keep you going. Support systems may come and go, the biggest and loyal support you have is in yourself. If you don’t believe in yourself or have the discipline to continue to push yourself and strive for greater, you will never gain success
***Is there anything in your sporting journey so far, that you would have done differently?***
Find better qualified people e.g. a coach earlier in my sporting career. (n-IP)
Worked with sports psych earlier (× 3 responses) (n-IP)
I would have spoken to more sports psychologists to manage the demands of my career on and off the diamond, to minimise breakdowns (N-IP)
Made the effort to find the right people to speak to when I missed on achieving goals (IP)
I wish I had asked for help sooner from professionals regarding my coping strategies and stress levels. The pressure and stress of hitting my peak in sport at a young age has permanently affected my mental and physical health and at the time I had severe effects on my mental health, weight and sleeping habits. I wish I had diversified my support network to outside of the sport itself. (n-IP)

### Psychological skills and attributes

Athletes were unanimous in perceiving psychological attributes as vital to their success, and all the psychological attributes previously reported by elite athletes were rated as highly important by more than 80% of both AB and AwD respondents across the achievement spectrum (Table 3) [3]. Notably, all IP-athletes stated that their *Ability to Change* and *Resilience* were vital to their success. Similarly, ‘*Self Discipline*’ and ‘*Mental Toughness’* were deemed as important by virtually all athletes. There were no statistically significant differences between males and females, or cgs and non-cgs athletes, for any of the responses concerning ‘Psychological Skills and Attributes’, but AB-athletes were more likely than AwD to perceive ‘*Ability to Manage Performance Nerves’* and ‘*Rituals’* as being vital to their success.

**Table 3:** Psychological Attributes: percentage of IP/n-IP and AB/AwD athletes who agree that these psychological attributes are vital to their success

Psychological Attributes	IP	n-IP	*p* value	AB	AwD	*p* value
Ability to adapt to change	100	94	0.053	96	95	–
Resilience	100	96	–	98	95	–
Self-discipline	98	100	–	99	100	–
Mental toughness	98	94	0.188	95	95	0.489
Ability to recover after injury	98	89	0.052	93	86	0.129
Being self-aware	98	91	0.099	93	95	0.362
Ability to manage emotions during competition	98	94	0.188	96	90	0.140
Goal setting and planning	95	94	0.392	94	95	0.423
Routines	95	87	0.190	90	86	0.272
Ability to manage performance nerves	95	91	0.232	95	81	0.012*
Strategic thinking	90	88	0.345	89	86	0.323
Reflection	90	90	0.500	91	86	0.222
Coping skills	90	90	0.500	90	90	0.484
Problem Solving	88	91	0.260	91	86	0.222
Breathing	88	80	0.156	85	71	0.063
Ability to create a supportive network	85	82	0.306	83	81	0.396
Being a knowledge seeker	83	78	0.264	78	86	0.226
Rituals	61	46	0.064	55	33	0.036*

*p < 0.05; **p < 0.01

These quantitative data were supported by the athletes’ written responses (qualitative data), whereby for example, when asked, “*If they achieved a medal at a benchmark event, why, in their opinion, did they succeed compared to their less successful counterparts?”* the athletes identified the themes of: superior self-regulation; strong mindset; and effective coping strategies as being fundamental.

Additionally, when in relation to psychological skills and attributes, they were asked for their thoughts and suggestions concerning “*What KEY advice/strategies would you advocate for younger athletes?”* the responses were in the themes of: Superior self-regulation; Intrinsic Motivation; and Effective Coping Strategies and Positive Mindset. Furthermore, when asked: *Is there anything in your sporting journey so far, that you would have done differently?* A key recommendation was: *“invested in mental training earlier”,* so as to develop ‘Superior Self-Regulation’ (IP).

### Interpersonal relationships (Table 4)

**Table 4:** Support network: importance, achievement classification (IP/n-IP), Able-Bodied/Athletes with a Disability classification, nature of the relationship and times of support

	Coach	Team-mate	Parents	Spouse/partner	Physiotherapist	Family members	Sports science Sports Medicine	Sports medicine Doctor	Sports psychologist	Sibling	Non-sporting Friend	Masseur
How important is this person in providing support? %	94	92	91	90	88	84	83	80	79	77	74	72
% Between achievement classification	IP: 100 n-IP: 93	IP: 97 n-IP: 91	IP: 97 n-IP: 88	IP: 96 n-IP: 88	IP: 97 n-IP: 84	IP: 90 n-IP: 81	IP: 94 n-IP: 76	IP: 89 n-IP: 74	IP: 89 n-IP: 73	IP: 82 n-IP: 74	IP: 90 n-IP: 68	IP: 92 n-IP: 61
% Between Able-Bodied/Athletes with Disability	AB: 97 AwD: 100	AB: 95 AwD: 100	AB: 95 AwD: 90	AB: 71 AwD: 62	AB: 84 AwD: 90	AB: 87 AwD: 100	AB: 65 AwD: 76	AB: 59 AwD: 65	AB: 63 AwD: 62	AB: 73 AwD: 76	AB: 76 AwD: 90	AB: 59 AwD: 76
With this person… (Top 2 highest rankings)	I depend on for my technical prowess Helps me find balance and perspective	I can let my hair down Helps me find balance and perspective	I share my worries and concerns Helps me find balance and perspective	I share my worries and concerns I don't discuss my sport with them	I depend on for my recovery strategies I depend on for my technical prowess	I don't discuss my sport with them Helps me find balance and perspective	I depend on for my recovery strategies I don't discuss my sport with them	I depend on for my recovery strategies I don't discuss my sport with them	I share my worries and concerns I don't discuss my sport with them	We can laugh together I don't discuss my sport with them	I can let my hair down Helps me find balance and perspective	I depend on for my recovery strategies I don't discuss my sport with them
This person provided support when… Top 1 highest ranking	During my early participation in sport	During my early participation in sport	During my early participation in sport	At senior elite level	At senior elite level	During my early participation in sport	At senior elite level	At senior elite level	At senior elite level	During my early participation in sport	During my early participation in sport	At senior elite level

Virtually all athletes (> 98%) perceived that having people to support them during their career was important for their success. These included: Family members/partners; Friends; Coaches and Professional Medical/Allied Heath staff, with all being highly prevalent in the athlete’s responses (72–94%). Furthermore, most (> 88% of athletes) said their coach’s ability to relate on a personal level was as important as their technical ability. Additionally, the majority of athletes (> 93%) agreed that having someone in their life who made them laugh was important to their success. The importance of family members was valued by more AwD (100%) than AB-athletes (87%) (*p* < 0.05), but there were no statistically significant differences between males and females, or cgs and non-cgs athletes in the responses to these questions.

### Performance factors/strategies

Mental preparation, recovery strategies, and using medical/allied health services were all rated as important, as was sleep (Tables 5 and 6).

**Table 5:** Recovery practices: percentage of IP and n-IP athletes, and AB and AwD athletes who practiced these techniques and procedure, statistical comparisons made using chi-square with contingency tables

Recovery practice	IP (%)	n-IP (%)	*p* value	AB (%)	AwD (%)	*p* value
Remedial massage	95	65	0.0001**	73	85	0.13
Stretching	90	84	0.173	86	85	0.44
Mindfulness	83	62	0.010*	70	67	0.396
Visualisation	80	67	0.064	74	57	0.061
Hot/cold immersion	76	50	0.004*	59	55	0.358
Ice Bath	76	54	0.012*	63	55	0.252
Hot/cold shower	74	61	0.080	62	81	0.052
Walking	71	69	0.433	79	25	< .001**
Meditation	66	50	0.049*	58	43	0.103
Plunge pool	59	27	0.0003**	38	35	0.401
Relaxation Massage	56	47	0.186	51	50	0.484
Yoga	51	36	0.058	44	30	0.131
Spa	46	44	0.416	50	20	0.007**
Cold water swimming	45	31	0.064	34	45	0.213
Pilates	37	25	0.086	28	30	0.444
Progressive muscle relaxation	34	37	0.396	37	29	0.226
Infrared sauna	20	4	0.002**	11	0	0.061
Finnish sauna	15	13	0.382	16	0	0.028*
Neuro linguistic programming	15	4	0.016*	4	25	0.001**
Dance	15	14	0.448	16	5	0.102
Intermittent hypoxic training	7	8	0.478	8	5	0.322
Floatation tank	5	7	0.298	7	5	0.38

*p < .05; **p < .01

**Table 6:** Athletes’ utilisation of service providers, their perceived effectiveness and referrals, comparisons made using chi-square with contingency tables

	Physiotherapist	Family GP	Masseur	Dietician/nutritionist	Sports psychologist	Sports Medicine Doctor	Acupuncturist	Chiropractor	Osteopath	Myotherapist	Naturopath/herbalist
% Utilisation	91	84	83	70	55	50	30	24	21	20	13
% Effectiveness	96	94	96	88	76	77	57	45	50	45	34
Referred by (top ranking)	State institute	Parent	Self/state institute	State institute	State institute	State institute	Self	Self/parent	Friend	Friend	Friend
% Utilisation by achievement classification (*p* value)	IP: 95 n-IP: 89 (0.113)	IP: 88 n-IP: 82 (0.217)	IP: 98 n-IP: 76 (0.001**)	IP: 85 n-IP: 62 (0.004**)	IP: 74 n-IP: 46 (0.002**)	IP: 76 n-IP: 38 (< 0.001**)	IP: 32 n-IP: 29 (0.370)	IP: 25 n-IP: 23 (0.230)	IP: 20 n-IP: 22 (0.411)	IP: 23 n-IP: 18 (0.278)	IP: 15 n-IP: 12 (0.335)
% Utilisation by able-bodied/with a disability (*p* value)	AB: 89 AwD: 100 (0.051)	AB: 82 AwD: 95 (0.064)	AB: 83 AwD: 85 (0.421)	AB: 70 AwD: 70 (0.5)	AB: 53 AwD: 67 (0.141)	AB: 49 AwD: 60 (0.175)	AB: 28 AwD: 39 (0.174)	AB: 27 AwD: 5 (0.02*)	AB: 22 AwD: 17 (0.306)	AB: 23 AwD: 6 (0.265)	AB: 16 AwD: 0 (0.037*)
% utilisation by gender (*p* value)	Male: 54 Female: 55 (0.861)	Male: 9 Female: 12 (0.611)	Male: 42 Female: 59 (0.047*)	Male: 16 Female: 19 (0.654)	Male: 12 Female: 20 (0.282)	Male: 2 Female: 11 (0.063)	Male: 2 Female: 6 0.334()	Male: 4 Female: 10 (0.243)	Male: 2 Female: 7 (0.207)	Male: 4 Female: 13 (0.097)	Male: 0 Female: 5 (0.136)

*p < .05; **p < .01

#### Recovery strategies

The most prevalent recovery strategies were: ‘Remedial Massage’, ‘Stretching’ and ‘Mindfulness’. ‘Visualisation’ was also highly prevalent, thereby further reiterating the perceived importance of psychological skills and attributes. Athletes’ perceived sleep, a lifestyle factor as well as being important for recovery, to be important and reported getting 7 ± 1 h a night. There were no differences between any of the athlete categories for the reported amount of sleep.

Eight recovery practices were identified with statistically significant differences (*p* < 0.05) in the prevalence of utilisation, with IP-athletes use being higher than n-IP-athletes for: Remedial Massage, Mindfulness, Hot/Cold Immersion, Ice-bath, Meditation, Plunge-pool, Infrared sauna, and Neuro Linguistic Programming (NLP). Finnish saunas and Spas were used more by AB-athletes than AwD (*p* < 0.05), and NLP used significantly more by AwD-athletes than AB-athletes (*p* < 0.005). There were no statistically significant differences between males and females, nor cgs and non-cgs athletes.

#### Service providers (Table *6*)

All athlete referrals to frequently used practitioners were via their State Institute, except the family GP. Overall, IP-athletes had higher utilisation than n-IP-athletes for all services except for osteopathy. Of these, there were statistically significant differences in the use of four service providers, with utilisation by IP-athletes being statistically greater (*p* < 0.05) for Massage Therapist; Dietician/Nutritionist; Sports Medicine Doctor; and Sports Psychologist. Chiropractors and Naturopaths were used significantly more by AB-athletes than AwD-athletes (*p* < 0.05) and Sports Psychologists were used significantly more by females than males (*p* < 0.05). There were no differences between cgs and non-cgs athlete.

#### Weight division practices

Around one quarter of participants (n = 35) were required to make a competition weight division (89% from combat sports). Nine athletes dropped 1–2 kg below what they considered their baseline training weight, 12 athletes dropped 5 kg below baseline, 6 athletes dropped 6-8 kg below baseline, and one athlete dropped 10 kg below baseline weight. Fifteen athletes reported using a sauna to lose weight before competing. Other weight-loss strategies included running, skipping, walking, and sports specific exercise. No differences in the prevalence of these practises were evident between; IP and non-IP; males and females, nor cgs and non-cgs athletes.

#### Qualitative responses

In response to the question: *If they achieved a medal at a benchmark event, why, in their opinion, did they succeed compared to their less successful counterparts?* Having the right support team was perceived to be crucial, particularly amongst IP athletes (IP 79%, n-IP 21%).

Responding to the question: *What could be done better to educate and empower athletes regarding their knowledge and effective usage of athlete and lifestyle strategies *etc*.*? Two new sub-themes emerged within Performance Strategies, these being: education (for athletes and coaches), which was mentioned by more than 98% of athletes, and get advice from experienced/retired athletes. Additionally, making effective use of sports science and sports medicine (SSSM), maximising training, and performance opportunities, were also emphasised.

### Lifestyle practices

The vast majority of athletes (> 91%) agreed that activities that helped them relax when not training or competing was important to their success. Similarly, Lifestyle practices such as: nutrition/hydration, the importance of having time-out, and lifestyle ‘balance’, were also perceived as important. As a recovery practice (mentioned previously), the importance of sleep was a common theme in recommendations athletes would make to younger athletes.

Athletes predominantly sought dietary advice from either a qualified naturopath or herbalist (95% IP 94% n-IP) or qualified dietician or nutritionist (44% IP 36% n-IP). The prevalence of source of dietary advice was not statistically significantly different between athlete categories. Most athletes (> 94%) predominantly ate home-cooked meals. A range of dietary practises were reported, with a ‘high protein’ diet being most prevalent, and the proportion of n-IP-athletes on a ‘high protein’ diet was statistically significantly higher than for IP-athletes (n-IP 54%, IP 38%) (*p* = 0.047). There were no other statistically significant differences in diet between IP and n-IP athletes and no statistically significant differences between the other athlete categories.

Water was the most common beverage consumed (> 95% of athletes). Teas and herbal teas were drunk by 20–30% of athletes, whilst the consumption of commercial sports drinks was only reported by 20% of athletes. The percentage of IP-athletes who regularly consumed coffee was significantly higher than for n-IP-athletes (IP 68% and n-IP 47%). There were no other statistically significant differences between athlete categories.

## Discussion

Psychological skills and attributes were perceived by athletes as vital to success across the range of attainment levels, with an ability to change and resilience being rated the highest. While the importance of resilience and adaptation is well documented [2], most athletes wished they had had access to these skills earlier in their career [2]. It may therefore be suggested that an holistic psychological support program, inclusive of training in mental fortitude [2], emotional intelligence [11], mental toughness [12], and mindfulness-acceptance-commitment (MAC) practices could be incorporated into athlete support programs to facilitate athlete wellbeing [13–15]. The suggested potential value of such an inclusion would be in assisting athletes to enhance performance and embrace the pressure and expectation to perform and succeed at important benchmark events, as well as contributing to the attainment of important psychological and mental aspects of wellness.

In support of this, athlete responses indicated that pre-elite athletes desired deeper knowledge, understanding, and autonomy. Given the focus on psychological skills being perceived to be critical to performance, this receptiveness presents an opportunity to hone and develop psychological skills earlier, so that they are well-practiced by the time the athlete reaches career maturity. However, interestingly, the second most common response concerning sports psychologists was: ‘*I don’t discuss my sport with them’.* Hence whilst the athletes’ perceived their sport psychologist to be important to them, their role may not always be what the referring Sports Governing Bodies and Institutes assume. Hence the potentially important role of ‘sport psychologists’ in contributing to the mental wellness of athletes should not be underestimated, and further clarification is required to better understand the multifaceted role of sport psychologists in providing support, education and skills training in both the athlete wellbeing/mental arena as well as for performance optimisation. Additionally, education about this service earlier in the developmental pathway may provide more targeted as well as holistic and effective use of this professional service, as indicated in the following quote:

I wish I had asked for help sooner from professionals regarding my coping strategies and stress levels. The pressure and stress of hitting my peak in sport at a young age has permanently affected my mental and physical health….I wish I had diversified my support network to outside of the sport itself. (n-IP-athlete).

All athletes perceived interpersonal relationships as vital, and valued people who made them laugh and supported them during their careers. Most also valued their coach’s ability to relate to them on a personal level as being as equally important as their technical ability. Perceived support is understood to affect emotional and informational esteem positively and have stress-buffering effects on self-confidence and resilience [2, 16, 17], including performance factors [18]. The coach-athlete relationship can directly influence an athlete’s motivation as the coach’s ability to facilitate autonomy-supportive behaviours, beneficially impact an athlete’s intrinsic and extrinsic motivation, drive performance and perseverance [17]. Conversely, in an environment where there is pressure to perform and high stress levels, some negative relationship aspects may develop, such as controlling behaviours, accidental violence [19], non-accidental violence, maltreatment, harassment, coercion, and abuse, which are not uncommon in elite sport [20, 21]. Hence an emphasis on a culture of personal support and respectful interpersonal relationships may not only serve to drive higher levels of performance and enhance athletes lives both within and external to the sporting arena but may also prevent potentially harmful interactions [4].

The results revealed that IP-athletes were more likely to utilise support services for recovery practices (hot/cold immersion, infrared sauna etc.), massage therapists, dietician/nutritionists, sports medicine doctors and sports psychologists. This finding concurs with our previous research, in which the use of ice-baths was reported as a common practice amongst world-class athletes and the finding that the use of hot/cold immersion [3], plunge-pools and ice-baths was more common amongst IP-athletes suggests perceived merit in introducing contrast bathing to athletes earlier in their career. Furthermore the current reported difference in the use of the aforementioned range of recovery practices is likely a reflection of access to facilities and professional services, since they are often recommended by NIN/NSO’s who also offer financial support. With the perceived benefit of these practises and services being indicated by numerous athletes who expressed that they would have liked access to these services earlier in sporting careers.

Further insight into athletes’ perceptions were gained from the responses to the qualitative questions, which resulted in the generation of two new sub-themes within the Performance Strategies theme, these being: education (for athletes and coaches), which was mentioned by more than 98% of athletes, and get advice from experienced/retired athletes. Within these was the advice to young athletes, which included being proactive in the context of being a knowledge-seeker, and ‘standing up for yourself’. Contextually this included, the advice to ‘change clubs earlier’. As some athletes commented that their choice to remain at their club was often due to loyalty or ‘aiming to please’ rather than making the right career choice. But with hindsight they reflected that it was important to know when to move to a more specialised club or DTE. Furthermore, the recommendations to young athletes included seeking professional assistance, such as sports psychologist, dietician and other specialists earlier, which reiterated the established athletes perceived benefits of these support services. Within these responses, the perceived importance of an understanding of nutritional strategies and the implementation of recovery strategies was strongly reflected, particularly concerning preventing burn-out, fatigue, and injuries.

Finally, a new lifestyle sub-theme of ‘balance’ emerged from the survey responses and was interlinked with social activity and connection/support, where athletes made recommendations to connect with other aspects of life such as study, family, and friends.

In virtually all aspects of the data there were almost no statistically significant differences between AB-athletes and AwD, and the few statistical differences that were identified may be due to their disability e.g., ‘walking’ was utilised more as a recovery practice amongst AB athletes (79%) compared to AwD (25%). Family support was greater for AwD, perhaps indicating support for managing various disabilities. The reasons why AB athletes reported practicing more rituals than AwD, requires further investigation as the reasons for this are not overtly evident. Again, there were very few statistically significant differences between males and females, but notably there were differences in the use of some recovery strategies and training hours leading up to and during competition.

## Conclusions

The findings of this study indicate that athletes of state level and above perceive lifestyle factors, performance strategies, and social support as important factors in sporting success. Their responses also indicate that they would welcome the implementation of educational strategies, recovery practices, and access to service providers earlier in their athletic career. To facilitate this, athlete development programs may benefit from facilitating athletes’ social support networks and implementing specific programs to foster a culture of respectful interpersonal relationships amongst athletes and their friends, coaches, family and support staff.

The importance of Psychological Skills and Attributes, Interpersonal Relationships, Performance Factors/Strategies, and Lifestyle Practices were all identified as important by the athlete, and where possible should be considered within holistic models of athlete development and support. Furthermore in such models there needs to be an awareness that the impact of the ‘sport psychologist may extend from aspects of sport performance into the broader realm of athlete well-being.

In conclusion, whilst athletes’ perceptions of the perceived benefits of services and lifestyle factors does not prove their significance in contributing to improved athletic performance and success, the perceptions of successful and aspiring athletes are nevertheless important in providing insight into their expectations of which support services and attributes are needed to most effectively enable their success, Furthermore it is recognised that athletes are individuals and their needs and expectations will vary, as indeed will the relative importance of the attributes and services between the diversity of athletic events/sports with their disparate physical, mental, and technical demands. Nevertheless, sporting institutions, coaches, support providers and athletes should be aware and informed of these potential factors, to thereby facilitate the most effective support for and development of athletes at the elite and pre-elite level.

## Data Availability

All data are securely stored on RMIT University servers in accordance with the approved ‘data management plan’. Collated data files may be accessed upon request.
